# Case Report: Unusual oral cavity changes associated with methamphetamine abuse

**DOI:** 10.3389/fpubh.2025.1473584

**Published:** 2025-04-02

**Authors:** Maksym Skrypnyk, Roman Skrypnyk, Tatiana Petrushanko, Margarita Skikevych, Vladymyr Petrushanko, Igor Skrypnyk

**Affiliations:** ^1^Department of Therapeutic Dentistry, Poltava State Medical University, Poltava, Ukraine; ^2^Department of Internal Medicine №1, Poltava State Medical University, Poltava, Ukraine; ^3^Department of Surgical Dentistry and Maxillofacial Surgery with Plastic and Reconstructive Surgery of Head and Neck, Poltava State Medical University, Poltava, Ukraine

**Keywords:** case report, methamphetamine, drug abuse, meth mouth, black hairy tongue, pulp necrosis, pityriasis rosea, palmar erythema

## Abstract

Methamphetamine abuse is a growing global health concern, recognized for its highly addictive properties and severe effects on the human body. Commonly referred to as crystal, chalk, or ice, methamphetamine is a synthetic stimulant that can be administered in various ways. Methamphetamine abuse is associated with a spectrum of oral health issues known as “meth mouth,” including rampant teeth caries, extensive occlusal tooth wear, periodontal diseases, xerostomia, bruxism, and poor oral hygiene. Despite the significant oral health impact, the exact pathogenesis remains unclear due to the limited number of reported cases and comprehensive studies performed. This case series details changes in oral and general health of different severity associated with methamphetamine abuse, highlighting unusual presentations such as the generalized decrease in teeth sensitivity, which can be associated with aseptic tooth pulp necrosis, hairy black tongue, rampant arrested caries, decreased periodontal inflammation, specific sunflower seed abrasions on antagonistic central incisors, pityriasis rosea skin lesion and palmar erythema. The clinical management was presented in detail and justified, which entails conservative dental, periodontal and oral mucosae treatments and highlighted the need for a comprehensive complex examination of these patients and financial consideration in treatment planning. This case series underscores the need to recognize the diverse oral and general health effects of methamphetamine abuse, which vary with duration and individual symptoms. Patients often withhold substance use, leading to delayed diagnosis until severe manifestations arise. Enhanced awareness among healthcare providers can improve diagnosis and management, offering valuable insights into underlying mechanisms and enabling better care for this high-risk population.

## 1 Introduction

Methamphetamine (METH) also called crystal, chalk or ice is a highly addictive psychostimulant that can be administered orally, smoked, snorted or injected (1). METH is the most commonly manufactured amphetamine-type stimulant globally (2). According to the latest United Nations Office on Drugs and Crime report, METH abuse is the third most common worldwide drug use disorder after cannabis and opioids (3). Unlike plant-derived substances, METH is a synthetic drug that does not require the extraction of active constituents from cultivated plants, allowing it to be manufactured almost anywhere (4). METH abuse has been recognized as a significant public health issue globally, in the United States data indicates that 59.7 per 1,000 adults have ever tried METH, and between 2015 and 2018, 6.6 per 1,000 adults used it in the past year. Among those, 52.9% developed METH use disorders (5). The prevalence of METH abuse, along with the incidence of overdose and mortality, has been increasing (6).

METH’s high lipophilicity enables it to readily cross the blood–brain barrier (7). Once in the brain, METH’s structural similarities with monoamines allow it to be recognized as a substrate by dopamine, serotonin, and noradrenaline plasma membrane transporters, facilitating its entry into neurons and their terminals (8). In the terminals, METH induces the release of monoamines from the monoamine storage vesicles, which significantly increases the concentration of monoamines in the synaptic cleft and with time causes the depletion of neurotransmitters from the monoamine storage vesicles (9). Additionally, METH inhibits monoamine oxidase, which in normal conditions provides monoamine degradation and blocks the reuptake of monoamines from the synaptic cleft which further increases their concentration in the synapse (10). After administration, patients experience an acute, powerful rush, an enhanced energy level, and prolonged euphoria (11).

Chronic METH abuse has systemic effects, including central nervous system stimulation, which can lead to anxiety, paranoia, hallucinations, and insomnia (1). Cardiovascular effects include tachycardia, hypertension, and increased risk of myocardial infarction and stroke (12). Chronic use can cause neurotoxicity, leading to cognitive decline and memory impairments (13). Other impacts include weight loss, malnutrition, and immune suppression, which heighten infection risks (1, 12). Behavioral effects include aggression and compulsive behaviors (14).

METH is also known to have numerous deleterious effects on oral health, collectively referred to as “meth mouth.” This term describes the severe dental caries, tooth wear, and periodontitis commonly observed among METH users (15). Similar to early childhood caries, METH-related caries predominantly affects the interproximal and vestibular surfaces of teeth, particularly the anterior teeth, resulting in a blackened, stained, rotting, crumbling, or disintegrating appearance (16). Additionally, individuals with METH abuse have higher incidences and severity of dental erosions, gingival enlargement, mucosal ulceration and tooth loss (17–20), xerostomia, periodontal diseases, clenching, and bruxism (18, 21–24), as well as neglecting of individual oral hygiene (25, 26).

The exact pathogenesis of METH’s effects on oral health is not fully understood, partly due to the relatively small number of reported cases (27). A more comprehensive understanding of the clinical manifestations and mechanisms by which METH influences oral health would improve diagnostic and treatment interventions for this group of patients.

The present case series aimed to present a unique variety of oral cavity changes in two patients after METH abuse, including rampant arrested caries and relatively decreased inflammatory response from periodontal tissues in addition to implemented treatment.

## 2 Case series description and case management

### 2.1 Patient 1

The first patient is a 35-year-old Caucasian male, who complained about an unesthetic appearance of his frontal teeth. He is not a regular dentist attendee. According to the patient, the destruction of his teeth started 3 years ago, and for the last 5 years, he has never experienced tooth pain or any other reasons to visit a dentist. After a few leading questions, the patient confessed that he regularly engaged in oral ingestion (by rubbing the crystals into his gums) and inhalation of METH regularly for about 3.5 years. He has been smoking cigarettes for 8 years (7–8 cigarettes daily) and smokes cannabis occasionally. Apart from heavy drug abuse in the past, he does not report any chronic systemic diseases or serious surgical interventions in the past.

**Figure 1:**
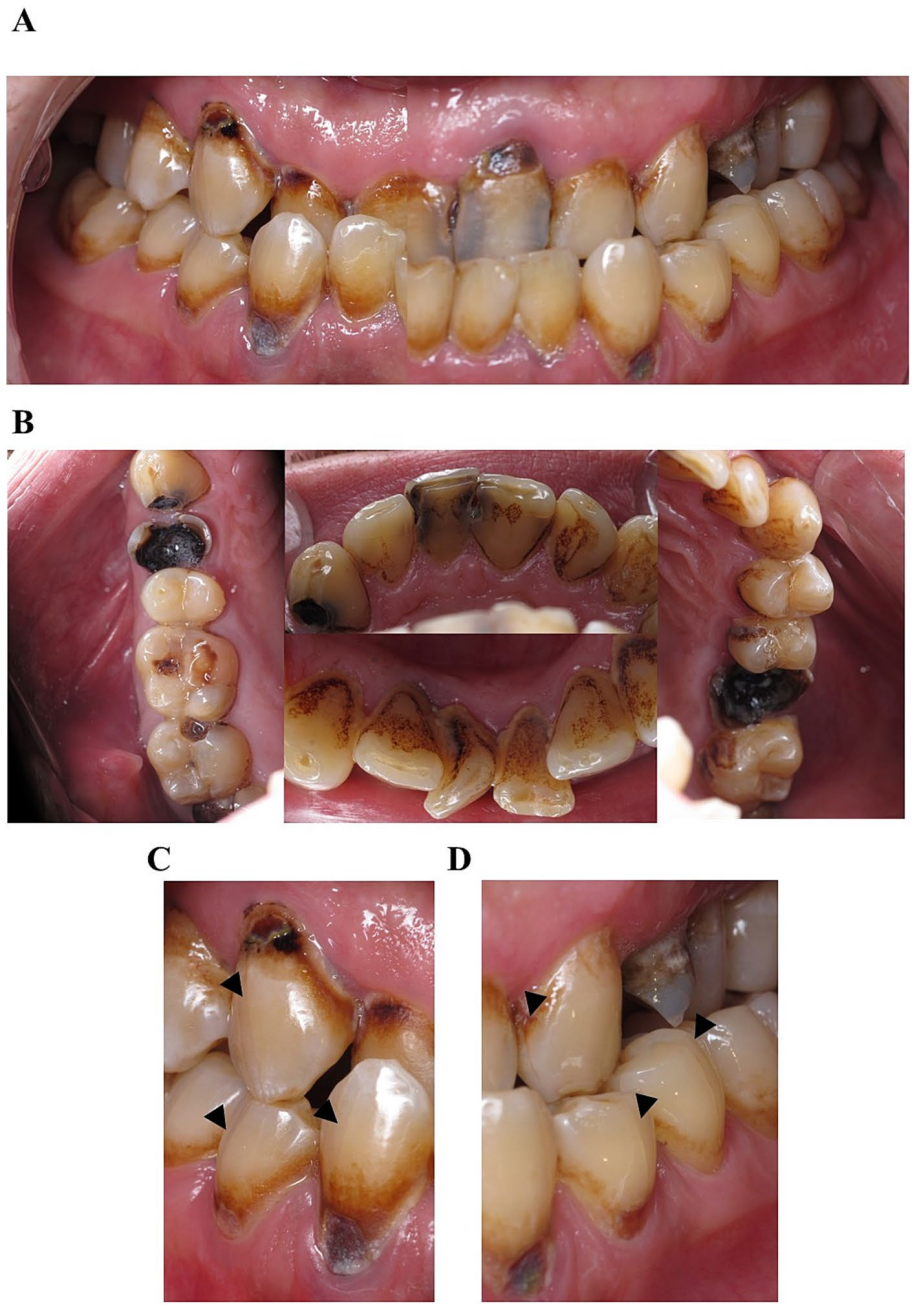
Clinical presentation of the first patient before treatment. Extensive and widespread caries affect multiple teeth, severely destroyed teeth, and poor oral hygiene. **(A)** Two separate images of the 1st and 4th sextants, and the 2nd and 3rd sextants, combined to illustrate the relationship of the teeth in central occlusion, **(B)** combined photo of the occlusal view of the first, second, third and fifth sextant, **(C)** vertical craze lines on right canines and premolars, denoted by black arrowheads and **(D)** the left side.

Upon intraoral examination, rampant caries of teeth and severe destruction of teeth 18, 28, 24, and 16 were diagnosed (Figures 1A,B). Multiple vertical craze lines were observed on the vestibular and oral surfaces of the teeth (Figures 1C,D). Teeth 11 and 41 had a notch-shaped defect on the incisal edge. Oral and vestibular surfaces were extensively covered with plaque (plaque score 100%), gingiva probing depth did not exceed 3 mm, bleeding on probing was 50%, and multiple recessions of gingival margin were present (Figure 1A). The patient’s oral mucosa was moderately moistened, with normal salivation observed. Regional lymph nodes were not enlarged and not painful on palpation. Based on the intraoral clinical examination, life and medical history the patient was diagnosed with multiple caries lesions of teeth (rampant caries) associated with METH abuse, chronic apical periodontitis of the tooth 16 and 24, chronic generalized gingivitis, multiple gingival margin recessions due to cervical and root caries, Angle Class III malocclusion, skeletal form with a reverse deep overbite and moderate crowding in the anterior segment of the upper and lower jaw.

To proceed with the treatment plan, the patient was advised to have a consultation with a general doctor and orthodontist, as well as a CT examination of the jaws. A general doctor examination did not reveal any significant systemic conditions. Following a consultation with the orthodontist, the patients agreed on orthodontic treatment involving fixed appliance braces, aimed at improving esthetics through “orthodontic camouflage.” After the analysis of a patient’s CT together with the orthodontist was decided to extract the hopeless tooth 16 and four wisdom teeth, that were severely destroyed by caries and restore the affected by caries molars, premolars, canines and mandibular incisors permanently and put provisional restoration on maxillary incisors, which will be changed for ceramic crowns after the orthodontic treatment will be completed with the creation of sufficient place and favorable occlusion. All other teeth were to be restored with direct composite restorations using the biomimetic technique, as detailed by Skrypnyk and Petrushanko (28). For all restoration that were made in this cases series following dental materials were used: Prime & Bond Universal adhesive (Dentsply Sirona, United States), SDR flow+ (Dentsply Sirona, United States), dentin - Spectrum TPH3 A3.5-O (Dentsply Sirona, United States), and for the main enamel – Neo Spectra A2/A3/A3.5 (Dentsply Sirona, United States). Direct composite restorations offer significant benefits in this clinical case, as they allow for adjustments after orthodontic treatment by adding composite material to the existing restorations.

The treatment plan was structured to be completed over seven appointments. During the first appointment, a thorough professional oral hygiene was performed, which entailed the removal of all dental deposits. The patient was also given individualized instructions on oral hygiene and diet. In each subsequent appointment, the treatment of one sextant was completed.

**Figure 2:**
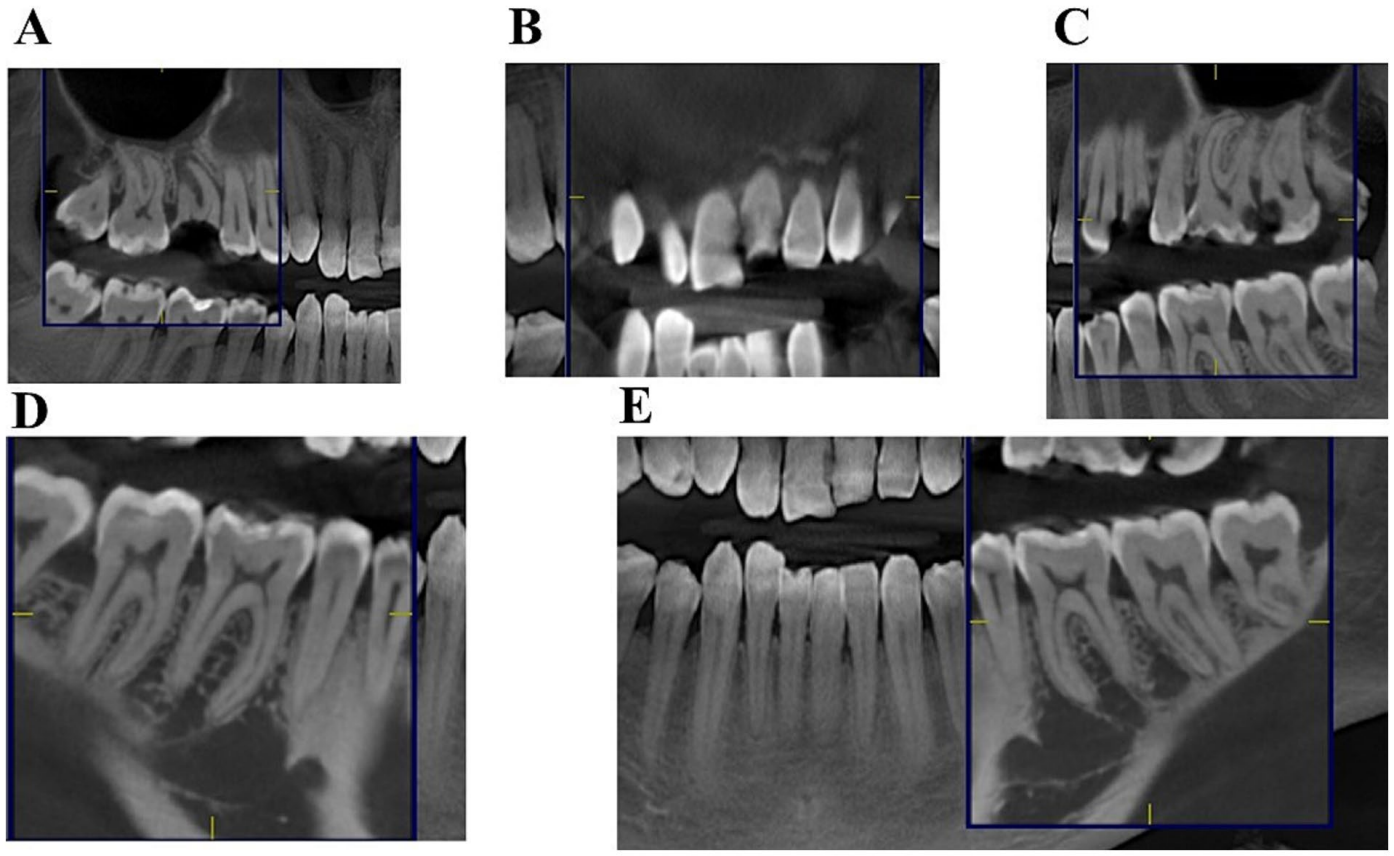
Patient 1. CT scan examination of the upper and lower jaw. A CT scan image of the **(A)** first sextant, **(B)** second, **(C)** third, **(D)** sixth, and **(E)** fourth and fifth sextants.

Before initiating caries treatment, tooth vitality was assessed with a cold test (Endo ICE, Roeco) and a heat test using heated gutta-percha (29). Surprisingly, all teeth in both the upper and lower jaws exhibited reduced or no response, even those with deep cavities. CT evaluation revealed no signs of apical periodontitis except for teeth 16 and 24 (Figures 2A,C). Clinically, all cavitated caries lesions resembled arrested lesions, characterized by densely covered with dark softened dentin, with sclerotic underlying dentine, with decreased sensitivity across all affected teeth, and did not have radiological signs of vitality loss (Figure 2). Carious cavities were prepared using a selective approach to remove affected tissue. Failure to determine tooth vitality using cold and heat tests necessitated the use of a cavity test (tooth preparation without anesthesia). Local infiltration anesthesia was administered to anesthetize the gingiva before placing the rubber dam clamp.

**Figure 3:**
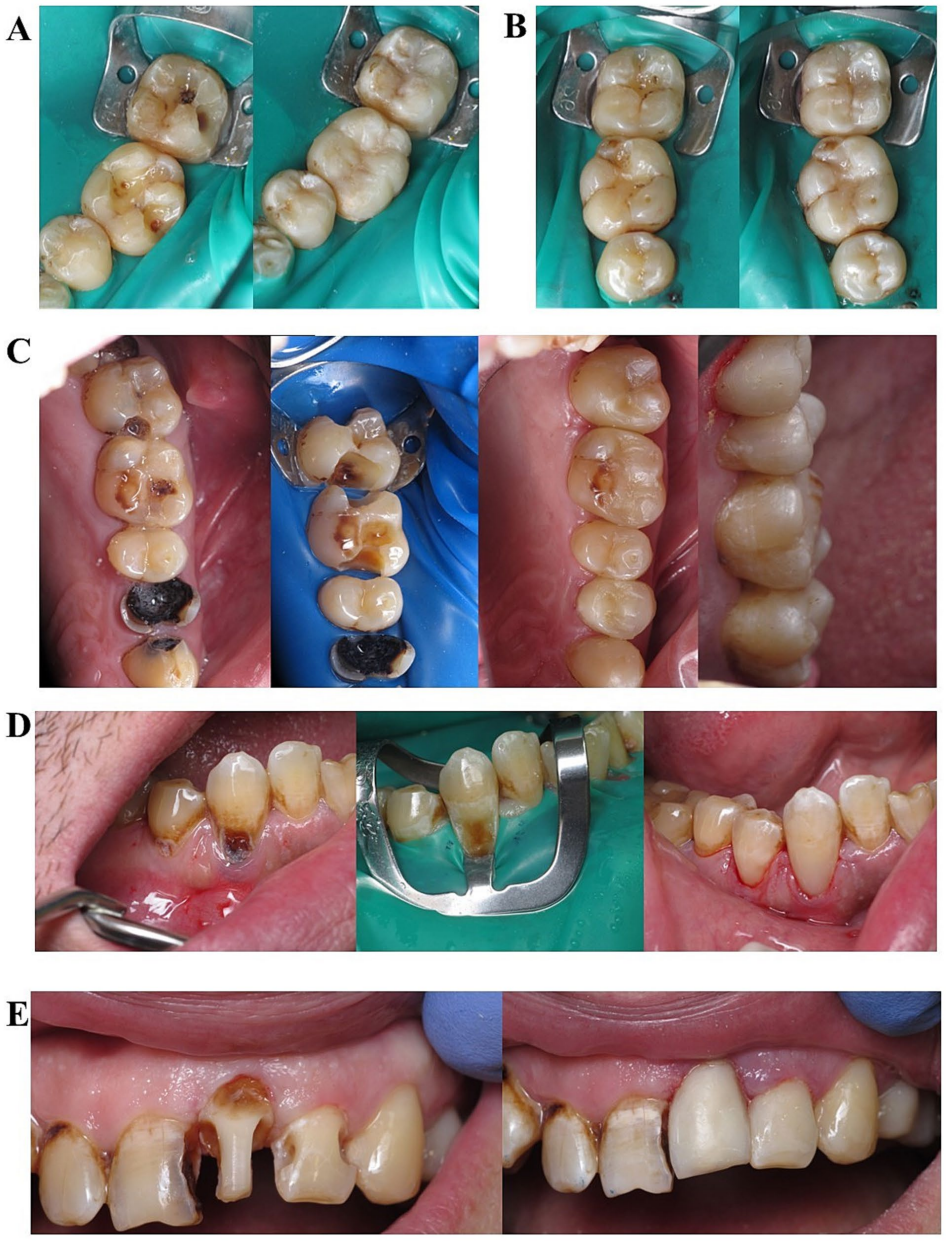
Treatment stages of the first patient. **(A)** Fourth and **(B)** sixth sextants (on left side teeth after selective carious tissues removal and on the right side teeth after direct restoration), **(C)** the combined image which illustrates stages of the third sextant restorative treatment: initial condition, teeth after selective carious tissues removal, occlusal and vestibular view of teeth after restoration (images presented from left to right), **(D)** the combined image which illustrates stages of the tooth 44 restorative treatment: initial condition, teeth after selective carious tissues removal, vestibular view of tooth 44 after restoration (images presented from left to right), **(E)** teeth 21 and 22 after selective carious tissues removal (left image) and after their direct restoration (right image).

In the upper right sextant caries cavities in teeth 17, 15, and 14 were restored with composite resin, while teeth 18 and 16 were extracted. Teeth 18 and 16 were extracted under greater palatine and posterior superior alveolar nerve block using articaine (Artifrin-Zdorov’e forte; 1:100000). The healing of the extraction sites was uncomplicated. In the upper left sextant, multiple carious cavities in 27, 26, and 25 teeth were restored with composite resin (Figure 3C). Tooth 24, which was severely destroyed by caries with an exposed pulp chamber, underwent root canal treatment and was restored with composite resin (Figure 3C). During endodontic access preparation, carious tissues were removed, and dry necrotic remnants of the pulp were identified, though there was no significant inflammatory response from the apical periodontal tissues based on radiological and clinical presentation (Figure 2C) and tooth 28 was extracted. The lower left and right sextant teeth 37, 36, 35, 34, 47, 46, 45, and 44 (Figures 3A,B) were restored on two other subsequent visits and teeth 38 and 48 were extracted. Restoring the anterior sextants of the upper and lower jaws was difficult due to the presence of deep subgingival cavities and root caries (Figures 3D,E). The low anterior sextant was restored permanently, and for the upper sextant provisional restorations were performed, which will be changed for the composite crown after orthodontic treatment (Figure 3E).

### 2.2 Patient 2

The second patient is a 24-year-old Caucasian female, who complained of unpleasant sensations while swallowing caused by a black “lump” on the base of her tongue and bleeding upon brushing her teeth. The sensation appeared around 10 days ago and was gradually increasing. She tried to remove it by scrubbing with a tongue cleaner for a week, but it was unsuccessful. She is not a regular dental attendee. She confessed to having been regularly addicted to METH for 2.5 years (inhalation and oral ingestion). She has been smoking for 6 years, around 12–15 cigarettes daily and used to smoke cannabis occasionally. Apart from drug abuse, a patient does not report any chronic systemic diseases or serious surgical interventions in the past.

**Figure 4:**
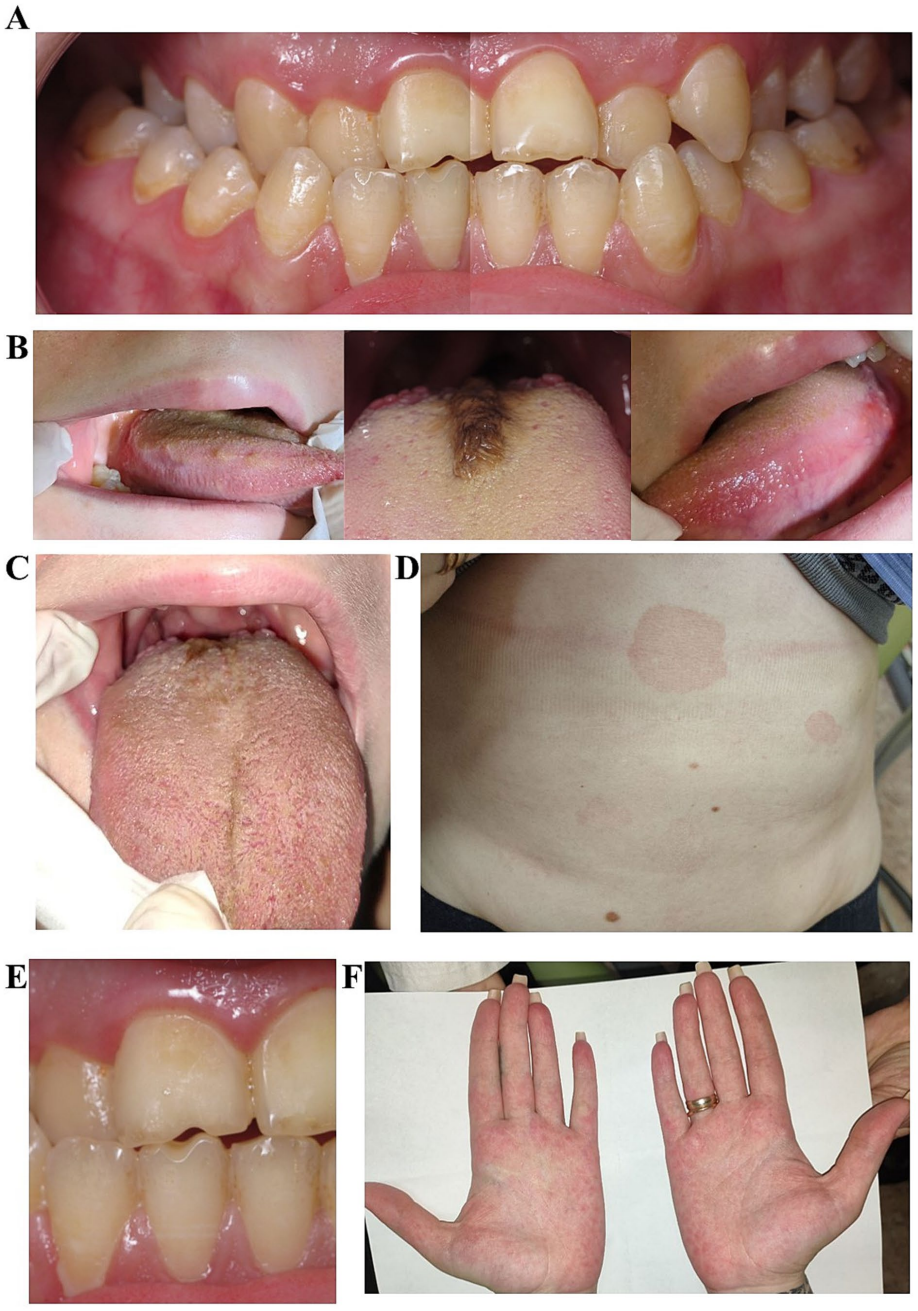
Clinical presentation of the second patient’s oral cavity before treatment. Notice, teeth are densely covered with dental plaque and the marginal gingiva is inflamed. **(A)** Two separate images of the 1st and 4th sextants, and the 2nd and 3rd sextants, combined to illustrate the relationship of the teeth in central occlusion. **(B)** Combined image that illustrates the view of the right lateral, dorsal (with black hairy tongue lesion), and left lateral surfaces of the tongue before treatment (from left to right), **(C)** clinical presentation of the dorsal surface of the tongue after treatment on a 10th day, **(D)** presentation of pityriasis rosea lesions on the skin of abdomen area, **(E)** higher magnification of teeth 11 and 41 teeth with a notch-shape abrasion defect on the incisal edge, caused by the shelling of sunflower seeds, **(F)** clinical presentation of the palmar erythema on a patient’s palms.

Upon intraoral examination, multiple sites of initial and cavitated carious lesions were diagnosed in teeth 16, 26, 35, 36, 37, 46, and 47 (Figure 4A). This patient had a similar notch-shaped defect on the incisal edge of teeth 11 and 41 as the first patient (Figure 4E). The oral and vestibular surfaces of teeth were extensively covered with dental plaque (plaque score 100%), gingival probing depth was 1–3 mm, and bleeding on probing was 93%. In the middle of the dorsal surface of the tongue base, a roller-shaped exophytic dark lesion was observed (Figure 4B). The skin on the palms exhibited a pattern of redness and pale areas, resembling a rash (Figure 4F). On the body’s skin (neck and abdomen area) oval-shaped skin rush with patches of different sizes (0.4–5 cm in diameter) were diagnosed (Figure 4D). Based on the intraoral clinical examination and the patient’s dental and medical history, a diagnosis was established for caries in teeth 16, 26, 35, 36, 37, 46, and 47, chronic generalized gingivitis, black hairy tongue (BHT), Angle Class III malocclusion, and moderate crowding in the anterior segment of the upper and lower jaw.

To proceed with the treatment plan, the patient was advised to undergo a general medical consultation, an orthodontic assessment, and an exfoliative cytology of the dorsal surface of the tongue. The general doctor did not diagnose any major somatic diseases, aside from palmar erythema and pityriasis rosea, no significant disturbances were found in the patient’s complete blood count and biochemical blood analysis, she also was negative for HIV, hepatitis B and C, and syphilis and had normal results of abdominal organs ultrasound examination. The exfoliative cytology from different parts of the oral mucosae showed abundant *Staphylococcus* microflora of hard palatine mucosae. The dorsal surface of the tongue exhibited a high abundance of *Staphylococcus* spp. and *Actinomycetes* spp. Few leukocytes were observed in the high-power fields, and approximately 30% of epithelial cells of the superficial (cornified layer) and granular layer were anucleated.

The following treatment plan was established: professional oral hygiene, diet analysis, further examination and treatment of palmar erythema and pityriasis rosea with the general practitioner, orthodontic consultation, and treatment of all cavitated carious lesions. For the management of BHT, the patient was prescribed rinsing the dorsal surface of the tongue with a solution made by dissolving 1,000,000 IU of benzylpenicillin powder (Arterium, Kievmedpreparat) in warm water. This rinsing was to be performed for 5 min, twice daily, after individual oral hygiene, for 5 days. Additionally, regular tongue cleaning with a tongue scraper was to be done every morning, before the oral rinsing with benzylpenicillin.

The patient agreed on professional hygiene of the oral cavity, diet analysis and treatment of the BHT. However, due to financial constraints, the patient opted not to proceed with caries treatment or further treatment with the general practitioner. Professional oral hygiene was performed the following day, and the patient began the oral rinsing regimen. Diet analysis revealed that the patient primarily consumed processed foods, high-sugar food, and sweetened beverages regularly. The diet was revised, and the patient was advised to increase the consumption of solid, unprocessed foods to enhance mastication and stimulate epithelial desquamation. Additionally, it was recommended to reduce the intake of sweets and sugary beverages. We strongly recommended discontinuing smoking; however, the patient found it impossible to comply. By the fifth day, the condition of the BHT had resolved (Figure 4C).

## 3 Discussion

The clinical presentation of METH-associated changes in the oral cavity can vary depending on the duration and frequency of abuse, as demonstrated in these two cases. Only severe and chronic METH abuse has quite specific multiple caries lesions covered with dark necrotic dentin, erosions of enamel, attrition, and multiple craze lines secondary to muscle parafunction due to bruxism of masticatory muscles trismus (30, 31). However, rampant caries is not specific to METH abuse only, a similar presentation can be seen in patients with radiation caries, severe xerostomia and adolescent rampant caries (32, 33). Interestingly, both patients had specific notch-shape defects caused by sunflower seed shelling on the incisal edges of teeth 11 and 41, which can be a family or cultural trait, previously it was described by Rath et al. (34). The mechanism of dental abrasion from sunflower seed consumption involves a combination of mechanical forces and repetitive mechanical actions of chewing the hard, fibrous seed husks. This process causes repetitive friction, which leads to enamel wear, particularly on the incisors and premolars, as these teeth are most engaged in breaking the husks (34, 35). Additionally, sunflower seed husks can create micro-trauma to the enamel and adjacent soft tissues, which may predispose to further damage by acidic or abrasive dietary substances (34, 35). A comparative study of adult METH abusers and non-abusers showed that 67.8% of METH abusers did not visit a dentist in the past year, compared to 31.25% of non-abusers (36). METH abusers had two times higher coronal caries rate, which is 29, and an incidence of root caries that was nearly three times higher - 4.54 compared to non-abusers (36). The study did not report significant differences in salivation between the two groups (36).

This is the first instance of generalized decreased tooth pulp sensitivity observed in a patient with chronic METH abuse (case 1). Based on our observations, it remains uncertain whether pulp vitality is preserved in treated teeth or if the pulp underwent aseptic, asymptomatic dry necrosis, which did not result in changes to periapical tissue radiolucency. The pulpal cavity was not exposed during the carious cavities preparation and there were no clinical and radiological signs of pulpitis or apical periodontitis that is why root canal treatment was avoided in teeth with decreased sensitivity to all pulp vitality tests. It might be related to the general influence of METH, rather than being locally caused by the active caries process in large severely destroyed teeth. This is suggested by the observation that even teeth with small carious cavities showed decreased sensitivity. While enamel loss typically increases sensitivity, the exposure of underlying dentin and loss of nerve signaling in severely damaged teeth may reduce sensitivity in advanced cases. Prolonged vasoconstriction caused by METH reduces blood flow to oral tissues, impairing the ability of tissue and nerve endings to heal and remain viable, which with time could potentially blunt nerve response, reducing pain perception (37, 38). In our opinion, the major pathogenic mechanism involved in reduced teeth sensitivity is chronic METH-induced stimulation of the central nervous system, which may alter pain perception, diminishing the sensation of dental discomfort despite ongoing damage (39). The effect of METH on the brain and coronary arteries has been described, according to this mechanism, METH can potentially cause chronic vasoconstriction of the pulp vessels, disrupting the blood supply to the pulp and leading to tissue hypoxia and aseptic necrosis (37, 40). Few clinical studies reported severe disruption of jawbones vascularization in METH abusers, which caused osteonecrosis of the jaw, which can be a result of toxic evaporation while heating of the white phosphor (30, 41, 42). The mechanism behind the decreased sensitivity of tooth pulp and its possible vitality loss could be similar. However, there are no experimental or clinical studies to support the impact of METH on tooth pulp.

Decreased blood perfusion of salivary glands caused by METH results in reduced salivation, which causes xerostomia (43). It decreases the buffer capacity of saliva by reducing salivary bicarbonate concentration, which impairs the ability to neutralize acids in the oral cavity and dental plaque (44). At presentation, neither patient exhibited signs of xerostomia, as this condition is reversible in METH abusers upon cessation of METH. It can take up to 30 days to restore the salivation volume and takes less time to normalize pH of saliva (45). Both patients had poor oral hygiene, high dental plaque and tartar accumulation, especially in subgingival and interproximal areas that caused gingivitis development, which is typical for METH abusers (31, 46). Gingival bleeding and inflammation were decreased in both cases, which can be explained by vasoconstriction, decreased vascular density and suppressed angiogenesis caused by nicotine in chronic cigarette smokers (47–49). Professional oral hygiene resulted in a significant improvement in periodontal health.

BHT is a benign condition characterized by a discolored, hairy appearance of the dorsal tongue. It is commonly found among smokers with poor oral hygiene, and immunocompromised patients. BHT consists of two components: elongated filiform papillae and discoloration of the dorsal tongue (50). In our case, we believe several factors contributed to the development of a BHT. First, hyperkeratosis, METH users typically have poor nutritional habits, leading to a deficiency in essential vitamins necessary for normal epithelial cell turnover. Inadequate desquamation on the dorsal tongue, caused by an insufficient diet, results in the abnormal accumulation of keratin layers, known as hyperkeratosis (51). Hyperkeratosis usually occurs at the tips of the filiform papillae leading to their elongation to lengths between 12 and 18 mm (the normal length of a filiform papilla is approximately 1 mm; a diagnosis for hairy tongue is made if it ≥3 mm) and a width of 2 mm, resulting in a hair-like appearance (52). The elongation of the filiform papillae causes the trapping of microscopic food particles, which creates a suitable environment for bacterial growth combined with poor oral hygiene typical for METH users (26, 36, 53). Moreover, METH users tend to prefer sweet foods, carbonated beverages which create an environment rich in monosaccharides and lower pH, further promoting bacterial growth (26, 54, 55). The elongation of the filiform papillae, poor oral hygiene, monosaccharides-rich diet and xerostomia cause a thriving environment for porphyrin-producing anaerobic chromogenic bacteria and yeast which create a BHT appearance (56, 57). Additionally, the patient in our case report was a heavy tobacco user, which is a well-known risk factor for BHT (58). A complex intervention, including normalization of oral hygiene to reduce the total bacterial load in the oral cavity, oral rinsing with antibiotics against the detected bacteria and normalization of the diet, resulted in the resolution of BHT.

The presence of pityriasis rosea in our patient can be explained by METH-induced immune deficiency. There is growing evidence that METH suppresses and modulates the immune system (59, 60). METH has significant effects on both the innate and adaptive immune responses (61). It causes a reduction in the number of natural killer cells and dendritic cells, which significantly impacts the adaptive immune system thereby predisposing to certain diseases and infections, including pityriasis rosea (62, 63).

The etiology of palmar erythema in our patient is most likely multifactorial. Given the normal complete blood count and liver function test values, the primary identifiable risk factor is tobacco smoking. According to McArthur and Firkin smoking is a possible independent factor leading to the development of palmar erythema (64). Additionally, considering the female sex of our patient it is possible that palmar erythema could be caused by an excess of estrogens (65). However, the patient declined to have her free estrogen levels tested.

There are no medication approved by the Food and Drug Administration for the treatment of METH use disorder. However, behavioral interventions have shown promising results (66). Among these, the most effective to date is contingency management, a behavioral intervention which is grounded in the principles of operant conditioning and rewards positive behaviors. In contingency management, material incentives are provided contingent upon biological confirmation of drug abstinence (67). Participants in contingency management programs earn rewards, such as vouchers or gift cards, for meeting specific goals, such as passing drug tests or attending counseling sessions. These rewards are provided promptly, reinforcing the link between the desired behavior and the positive outcome. Contingency management has proven highly effective in encouraging abstinence from various substances, including alcohol, nicotine, opioids, and stimulants (68–71). A recent review by Asha Rani et al. analyzed seven studies evaluating the efficacy of contingency management in treating METH use disorder. All seven studies demonstrated a reduction in METH use, and two studies additionally reported improvements in other health-related behaviors (72). Overall contingency management is highly effective in reducing METH use and promoting attendance to recovery-related appointments in people with METH use disorders (67).

The second psychotherapy approach in management of METH use disorder is cognitive behavioral therapy, which aims to change patterns of thinking and behavior of METH users, while equipping individuals with skills to manage cravings and prevent relapse (73, 74). Several meta-analyses and randomized clinical trials have found that cognitive behavioral therapy effectively reduces METH use and improves psychosocial functioning when delivered consistently over time and is considered one of the most effective behavioral interventions for stimulant use disorders (75–80).

The third psychological treatment approach is motivational interviewing, an empirically supported clinical method designed to help individuals make behavioral changes to achieve personal goals. Using specific techniques, motivational interviewing empowers individuals to mobilize their intrinsic values and goals, exploring and resolving ambivalence about change (81). Motivational interviewing is often used in combination with cognitive behavioral therapy and has demonstrated high efficacy in reducing METH use and improving medication adherence (82).

While the first-line treatment for METH use disorder is psychotherapy, psychotherapy is not always available, which leads to a search for other treatment options (83). Additionally, the effectiveness of psychotherapy often diminishes after discharge (84). Bupropion and naltrexone used individually have shown positive evidence of efficacy in clinical trials for the treatment of METH use disorder (77, 85–88). Bupropion is a stimulant-like antidepressant that acts as a norepinephrine-dopamine reuptake inhibitor, enhancing the levels of dopamine and norepinephrine in the brain. Through the norepinephrine and dopamine systems, bupropion provides amelioration of the dysphoria associated with METH withdrawal that drives continued use (88, 89). Naltrexone is an opioid-receptor antagonist, which by blockage of mu-opioid receptors, can affect the reward pathways linked to addiction. Naltrexone is highly effective for the treatment of opioid use disorder and has modest efficiency in preventing relapse of alcohol use (90, 91). According to recent clinical trials a combination of extended-release injectable naltrexone combined with once-daily oral extended-release bupropion provided a higher treatment response than a placebo in people with METH use (88, 92). Overall, the combination of bupropion and naltrexone is not a cure, but it is a step forward in management of METH use disorder, for which effective pharmacological treatments were previously lacking.

Both patients experienced socio-economic challenges in accessing dental treatment, including limited financial resources and difficulty establishing trust with healthcare providers. METH users are primarily adults from low socio-economic backgrounds, which often creates a financial barrier to accessing essential and timely treatment (5, 36). METH users encounter additional social barriers in accessing healthcare services, which include challenges related to establishing trusted, continuous care, obtaining integrated support from multiple medical services, and overcoming judgmental attitudes from healthcare practitioners (93).

To summarize, changes in oral health in METH abusers are caused by a triad of factors: limited access to dental care due to financial burdens, negative attitudes toward their health, and the direct impact of drug use. The risk of oral diseases is heightened by initial changes in the oral mucosa, xerostomia, insufficient diet and poor oral hygiene, all of which compromise oral health. General practitioners and dentists should be aware of the manifestations of METH abuse in the oral cavity, as it has high diagnostic value and is easily accessible for examination. The cases presented demonstrate that METH abuse can have various manifestations in the oral cavity, ranging from mild to severe. Moreover, some patients may not openly discuss their substance use. Identifying the type of substance and the duration of abuse is crucial for effective treatment planning and substance abuse management. In patients where the dentist suspects substance abuse based on the oral cavity status, the most important thing is to make the patient feel comfortable and create a trusting atmosphere for the conversation. It’s crucial to avoid a stigmatizing or prejudiced attitude, as this can make patients feel unsafe, and ashamed, and may lead them to stop seeking treatment. Dentists can be among the first specialists to identify METH abuse and encourage patients to seek further treatment for METH dependence, helping to reduce the socio-economic burden of METH abuse and potentially save lives.

## Data Availability

The original contributions presented in the study are included in the article, further inquiries can be directed to the corresponding author.
