# Supplementary material for: Case Report: Unusual oral cavity changes associated with methamphetamine abuse
Source: Front Public Health. 2025 Apr 2;13:1473584. doi: 10.3389/fpubh.2025.1473584 (PMC12000065; doi:10.3389/fpubh.2025.1473584)
Supplement: Supplementary file 1 [file Data_Sheet_1.pdf]

CARE case report guidelines

CARE Checklist of information to include when writing a case report

(cc) BY-NC-ND

Topic	Item	Checklist item description	Reported on Line
Title	1	The diagnosis or intervention of primary focus followed by the words "case report"	55-60
Key Words	2	2 to 5 key words that identify diagnoses or interventions in this case report, including "case report"	96-98
Abstract (no references)	3a	Introduction: What is unique about this case and what does it add to the scientific literature?	70-78
	3b	Main symptoms and/or important clinical findings	78-84
	3c	The main diagnoses, therapeutic interventions, and outcomes	84-87
	3d	Conclusion—What is the main "take-away" lesson(s) from this case?	87-94
Introduction	4	One or two paragraphs summarizing why this case is unique (**may include references**)	163-166
Patient Information	5a	De-identified patient specific information.	172-176 307-310
	5b	Primary concerns and symptoms of the patient.	172-173 308-318
	5c	Medical, family, and psycho-social history including relevant genetic information	176-180 310-316
	5d	Relevant past interventions with outcomes	181-183 316-318
Clinical Findings	6	Describe significant physical examination (PE) and important clinical findings.	183-235 318-335
Timeline	7	Historical and current information from this episode of care organized as a timeline	236-302 336-374
Diagnostic Assessment	8a	Diagnostic testing (such as PE, laboratory testing, imaging, surveys).	183-249 318-335
	8b	Diagnostic challenges (such as access to testing, financial, or cultural)	388-390
	8c	Diagnosis (including other diagnoses considered)	430-686
	8d	Prognosis (such as staging in oncology) where applicable	N/A
Therapeutic Intervention	9a	Types of therapeutic intervention (such as pharmacologic, surgical, preventive, self-care)	236-259 336-374
	9b	Administration of therapeutic intervention (such as dosage, strength, duration)	375-384
	9c	Changes in therapeutic intervention (with rationale)	N/A
Follow-up and Outcomes	10a	Clinician and patient-assessed outcomes (if available)	387-424
	10b	Important follow-up diagnostic and other test results	N/A
	10c	Intervention adherence and tolerability (How was this assessed?)	281-302 387-424
	10d	Adverse and unanticipated events	N/A
Discussion	11a	A scientific discussion of the strengths AND limitations associated with this case report	430-851
	11b	Discussion of the relevant medical literature **with references**.	430-827
	11c	The scientific rationale for any conclusions (including assessment of possible causes)	430-827
	11d	The primary "take-away" lessons of this case report (without references) in a one paragraph conclusion	828-851
Patient Perspective	12	The patient should share their perspective in one to two paragraphs on the treatment(s) they received	N/A
Informed Consent	13	Did the patient give informed consent? Please provide if requested	Yes ☑ No ☐
